# The Role of the High-Sensitivity C-Reactive Protein in Patients with Stable Non-Cystic Fibrosis Bronchiectasis

**DOI:** 10.1155/2013/795140

**Published:** 2013-12-07

**Authors:** Meng-Heng Hsieh, Yueh-Fu Fang, Guan-Yuan Chen, Fu-Tsai Chung, Yuan-Chang Liu, Cheng-Hsien Wu, Yu-Chen Chang, Horng-Chyuan Lin

**Affiliations:** ^1^Department of Thoracic Medicine, Chang Gung Medical Foundation, Department of Chest Medicine, Chang Gung University, College of Medicine, Taoyuan 33342, Taiwan; ^2^Department of Radiology, Chang Gung Medical Foundation, Department of Chest Medicine, Chang Gung University, College of Medicine, Taoyuan 33342, Taiwan; ^3^Department of Nuclear Medicine, Chang Gung Medical Foundation, Department of Chest Medicine, Chang Gung University, College of Medicine, Taoyuan 33342, Taiwan

## Abstract

*Study Objectives.* The aim of this study is to investigate the correlation between serum high-sensitivity C-reactive protein (hs-CRP) and other clinical tools including high-resolution computed tomography (HRCT) in patients with stable non-CF bronchiectasis. *Design.* A within-subject correlational study of a group of patients with stable non-CF bronchiectasis, who were recruited from our outpatient clinic, was done over a two-year period. *Measurements.* Sixty-nine stable non-CF bronchiectasis patients were evaluated in terms of hs-CRP, 6-minute walk test, pulmonary function tests, and HRCT. *Results.* Circulating hs-CRP levels were significantly correlated with HRCT scores (*n* = 69, *r* = 0.473, *P* < 0.001) and resting oxygenation saturation (*r* = −0.269, *P* = 0.025). HRCT severity scores significantly increased in patients with hs-CRP level of 4.26 mg/L or higher (mean ± SD 28.1 ± 13.1) compared to those with hs-CRP level less than 4.26 mg/L (31.7 ± 9.8, *P* = 0.004). Oxygenation saturation at rest was lower in those with hs-CRP level of 4.26 mg/L or higher (93.5 ± 4.4%) compared to those with hs-CRP level less than 4.26 mg/L (96.4 ± 1.6%, *P* = 0.001). *Conclusion.* There was a good correlation between serum hs-CRP and HRCT scores in the patients with stable non-CF bronchiectasis.

## 1. Introduction

Despite improvements in childhood immunization and tuberculosis control, bronchiectasis remains a significant clinical issue worldwide [1, 2]. It is a chronic, debilitating lung disease characterized by irreversible dilatation of the bronchi from airway remodeling due to chronic airway inflammation and infection. Underlying etiologies include autoimmune diseases, severe infections, genetic abnormalities, and acquired disorders; however, its pathogenesis and progression remain poorly understood [1–5]. Exacerbations occur at rates of 1.5–6.5 per patient per year [6, 7] and are associated with an increased risk of admission and readmission to hospitals and high healthcare costs [8].

High-resolution computed tomography (HRCT) is a proven, reliable, and noninvasive method for assessing bronchiectasis [9]. It can accurately diagnose bronchiectasis and localize and describe areas of parenchymal abnormality. A link between morphological HRCT parameters and clinical functional correlation has been established [9–14]. However, concerns over radiation exposure and high cost limit its frequent use in stable bronchiectasis patients.

Inflammation in bronchiectasis is characterized by persistence and intensity. Airway inflammation is neutrophil-predominant, and inflammatory profiles show increased levels of proinflammatory cytokines such as IL-1, IL-6, and TNF-*α* and low levels of anti-inflammatory cytokines such as IL-10 [3, 15, 16]. Elevation of systemic inflammatory markers, such as C-reactive protein (CRP) and total white cell count, has been found to correlate with the extent of the disease and poor lung function [17]. CRP is a pentraxin structure composed of five 23 kDa subunits. It is highly stable and allows measurements to be made accurately in both fresh and frozen plasma, without requiring special collection procedures. Moreover, high-sensitivity assays for CRP have been standardized across many commercial platforms. The long plasma half-life of CRP (18 to 20 hours), stability over a long period of time, and almost no circadian variation make it an accurate and sensitive marker of low-grade systemic inflammation [18, 19]. While the use of hs-CRP in cardiovascular diseases has been documented [20–24], its role in stable bronchiectasis remains unknown. Thus, the aim of this study was to explore the relationship between hs-CRP and severity scores on HRCT and other clinical variables in stable non-CF bronchiectasis patients.

## 2. Methods

### 2.1. Study Population and Design

One hundred and twenty-five (125) patients with bronchiectasis were recruited from the Thoracic Outpatient Clinic of Chang Gung Memorial Hospital in Taiwan from January 2006 to December 2007. The inclusion criteria were as follows: bronchiectasis documented on chest HRCT, idiopathic etiology of bronchiectasis (none of the patients with background suggests cystic fibrosis such as chronic dysfunction of the pancreas or liver or intestine or an electrolyte imbalance, disease onset before adolescence, and family history), chronic sputum production (daily sputum ≥ 10 mL), absence of other major pulmonary diagnoses, and a steady state defined by the absence of changes in symptoms noted by the patient over the past 3 weeks. The exclusion criteria were as follows: bronchiectasis with defined etiology (i.e., primary ciliary dyskinesia and allergic bronchopulmonary aspergillosis), common variable immunodeficiency, and use of antibiotics within the last three weeks. Patients with hepatic failure, malignancy, or pregnancy were also excluded.

The study design was conducted with approval of the Institutional Review Board (IRB) of Chang Gung Medical Foundation (IRB no. 97-1105A3). All patients provided written informed consent to participate in this study. The methodology and patient confidentiality were also approved by our IRB.

### 2.2. Measurement of Serum High-Sensitivity C-Reactive Protein Levels

Blood was drawn for measurement of serum inflammatory markers. The blood samples were then centrifuged at 3000 rpm at 4°C for 15 minutes, and aliquots were stored at −70°C. A latex turbidimetric immunoassay with a sensitivity of 0.01 mg/L was used to measure circulating levels of hs-CRP (Biomedical Laboratory Inc.).

### 2.3. High-Resolution Computed Tomography (HRCT)

The scoring system for HRCT described by Brody was used, and a score sheet was completed for each lobe of the lung [25]. Briefly, each lung lobe (considering the lingula and middle lobe as independent) was scored as 0 (no bronchiectasis), 1 (cylindrical bronchiectasis in a single lung segment), 2 (cylindrical bronchiectasis > 1 lung segment), or 3 (cystic bronchiectasis). The maximum score for each lobe was 12 points and a single radiologist with five years of experience in thoracic CT interpretation assessed the HRCT images in random order, without clinical functional information. This scoring system was used in a previous study, with the bronchiectasis score ranging from 0 to 72.

Two experienced radiologists, both pulmonary division consultants with more than 5 years of experience, scored the HRCT of these patients without any clinical data information. The interobserver agreement was 0.946 (data not shown).

### 2.4. Six-Minute Walk Test (6 MWT)

The 6-minute walk tests using the standard protocol described in the 2002 American Thoracic Society (ATS) statement [26] were performed at the outpatient clinic visit by well-trained technicians with at least three years of experience in performing 6 MWTs. Pre- and posttest oxygenation saturation under room air, walking distance, and standard spirometry before the test were recorded.

### 2.5. Body Mass Index (BMI)

Height was measured with a rigid stadiometer, and weight was measured by a calibrated digital scale. Body mass index (BMI) was calculated by dividing the weight (kilograms) by the height (meters squared), and then the quotient was converted into age- and sex-adjusted percentiles based on population data from NHANES 2000.

### 2.6. Statistical Analysis

Data were presented as mean ± SD, and all statistical analyses were performed using SPSS version 13.0 (SPSS Inc., Chicago, IL, USA). Independent Student *t*-tests or chi-square tests were performed to compare the clinical parameters, as appropriate. For bivariate analysis, we stratified the participants, using a cutoff point of serum hs-CRP of 4.26 mg/L, into two groups according to previous exacerbation-related hospitalizations (less than 2 times versus 2 times and above). To compare hs-CRP with other clinical variables, we used age (years), BMI (kg/m^2^), FVC (L), FEV_1_ (L), FEV_1_/FVC, IgE (KU/L), ECP (mcg/L), rest O_2_%, lowest O_2_%, ΔO_2_%, 6-minute walking distance (6 MWD, meters), and HRCT scores for correlation analysis. Correlations between data were analyzed using Pearson's correlation tests. A *P* value of less than 0.05 was considered significant.

## 3. Results

### 3.1. Patient Characteristics

**Figure 1:**
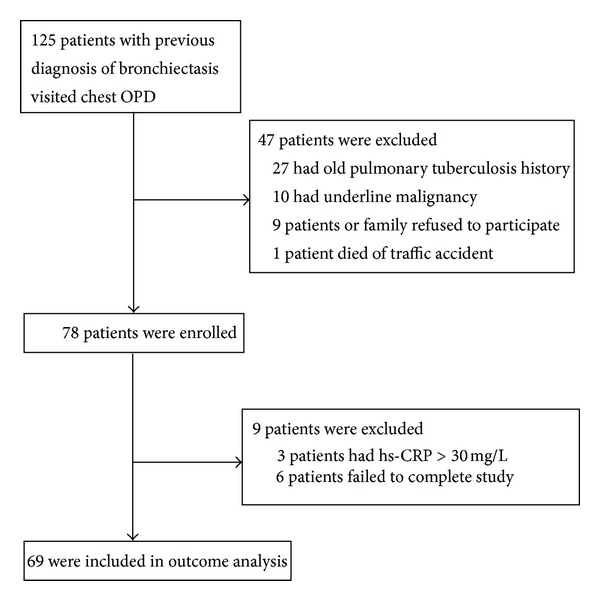
Flowchart of patients in the study cohort.

During the study period, 125 patients with bronchiectasis were recruited in the chest outpatient department, and 78 patients were evaluated for this study. Sixty-nine patients who met the inclusion criteria were enrolled in the study (Figure 1), and their demographic data are shown in Table 1. Three patients with a serum hs-CRP level of more than 30 mg/L without overt clinical symptoms or signs of infection at enrolment were excluded from the final data analysis because of the exacerbations and oral antibiotics given in the following weeks. Their serum hs-CRP levels were 45.51, 55.47, and 78.41 mg/L, respectively.

**Figure 2:**
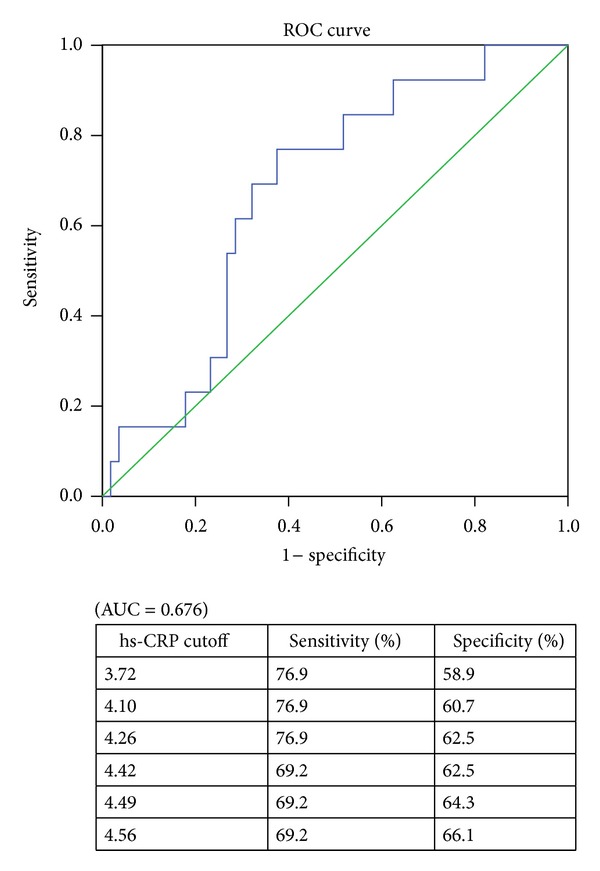
ROC curve of hs-CRP for prediction patients with repeated hospitalization (≥2 exacerbation-related hospitalization events).

Following the initial evaluation of hs-CRP, the patients were divided into two groups according to their previous exacerbation-related hospitalizations: those with an hs-CRP level less than 4.26 mg/L (*n* = 38) and those with an hs-CRP level of 4.26 mg/L or higher (*n* = 31) (Figure 2). We defined exacerbation-related hospitalizations as those with symptoms/signs of lower respiratory tract infection including cough, increased sputum production, changes in the sputum characteristics, hemoptysis, poor appetite accompanied by body weight loss, and need for further bronchodilator treatment, requiring treatment with systemic steroids or accompanied by respiratory failure. The characteristics and outcomes of the two groups of patients with stable bronchiectasis are shown in Table 2.

### 3.2. Circulating hs-CRP and Clinical Assessments

**Figure 3:**
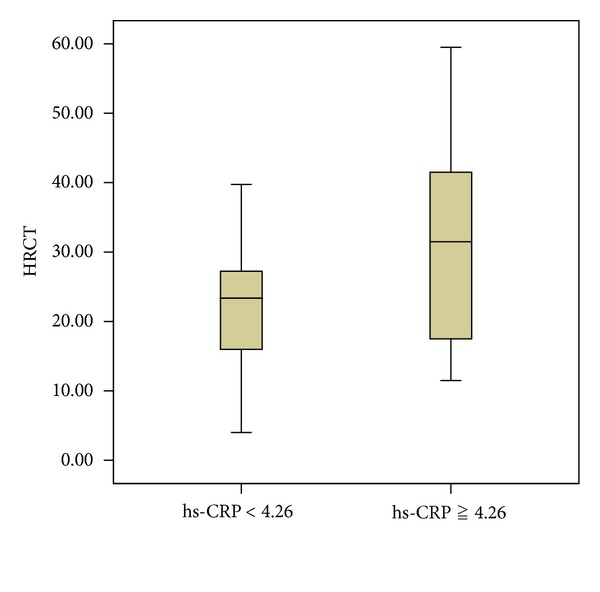
HRCT scores in higher and lower serum hs-CRP groups. HRCT scores were significantly higher in bronchiectasis patients with higher hs-CRP (mg/L). Boxes, median and interquartile range; whiskers, full range of values obtained; *P* = 0.004.

**Figure 4:**
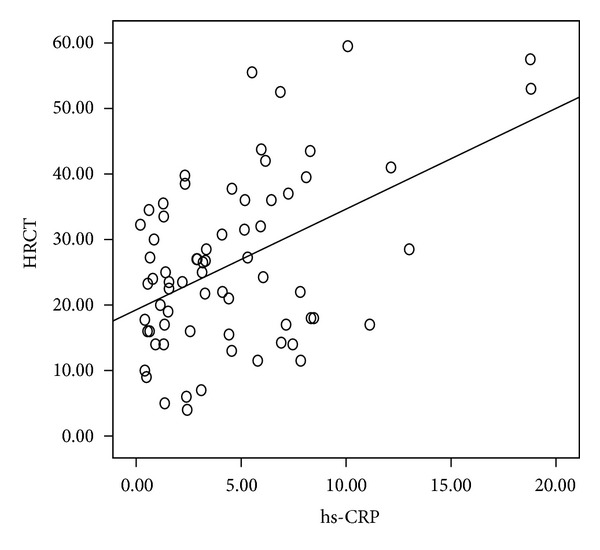
Relationship between serum high-sensitivity C-reactive protein (hs-CRP, mg/L) levels and HRCT scores in patients with stable bronchiectasis (*n* = 69, *r* = 0.473, *P* < 0.001, by Pearson's correlation).

**Figure 5:**
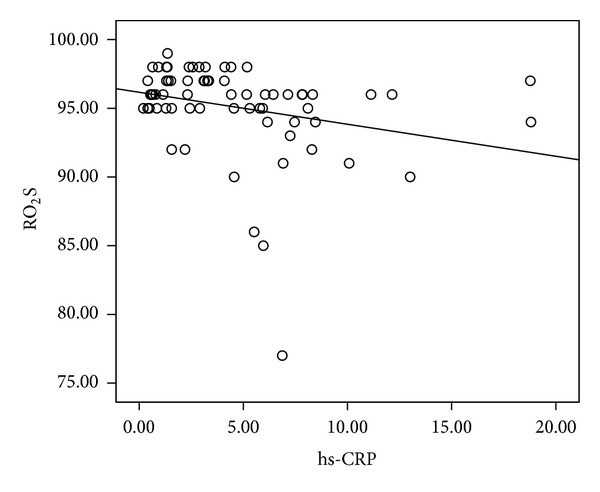
Relationship between serum high-sensitivity C-reactive protein (hs-CRP, mg/L) levels and RO_2_S% (rest oxygenation saturation under room air) in patients with stable bronchiectasis (*n* = 69, *r* = −0.269, *P* = 0.025 by Pearson's correlation).

There were no statistical differences in age, sex distribution, smoking status, BMI, pulmonary function test, and 6 MWT between the two groups. Bacteriology and regular treatment regimens (data not shown) were also similar. The HRCT scores were significantly increased in the higher hs-CRP group compared with the lower group (Table 3 and Figure 3). Resting oxygenation saturation was significantly decreased in the higher group than in the lower group, and there was a trend that patients with a lower hs-CRP had higher pulmonary functions (FVC and FEV_1_). Circulating hs-CRP levels were also significantly correlated with HRCT scores (*n* = 69, *r* = 0.318, *P* = 0.009) (Figure 4) and inversely correlated with rest oxygenation saturation (*r* = −0.349, *P* = 0.004) (Figure 5). The correlation between resting oxygenation saturation and HRCT score severity showed a moderately negative linear relationship (*P* < 0.001, *r* = −0.478, figure not shown).

## 4. Discussion

The pathogenetic mechanism leading to bronchiectasis is complex and still not well understood [1–5]. The current point of view considers that idiopathic bronchiectasis, chronic bronchial infection, and inflammation interact with each other leading to progressive lung damage [3]. The associated airway inflammation in bronchiectasis has been studied more widely recently; however, little is known about the intensity of low-grade systemic inflammation. To the best of our knowledge, this is the first study to describe the relationship between serum hs-CRP, rather than traditional CRP, and clinical variables including disease severity and HRCT in a group of patients with stable non-CF bronchiectasis. Our results demonstrated that hs-CRP had a good correlation with HRCT severity scores and may serve as a chronic inflammatory marker in the stable status phase of non-CF bronchiectasis patients, despite its broad clinical spectrum.

Progressive idiopathic bronchiectasis includes at least two subsets of patients [2, 3]. One subset, which constitutes the vast majority of cases, deteriorates over decades with an increased frequency of exacerbations, sputum volume, and extent of bronchiectasis. The other subset, usually those with single-lobe involvement, can be asymptomatic between exacerbations or without overt exacerbations and does not deteriorate even after decades. However, little is known about the severity and disease activity. No study has yet assessed the severity and disease activity of idiopathic bronchiectasis, even though two long-term studies have assessed the factors influencing mortality [27, 28].

C-reactive protein is predominantly produced in the liver, and IL-1, IL-6, and TNF-*α* have been identified as regulators of its production [29, 30]. More sensitive immune assays for CRP (high-sensitivity CRP, hs-CRP) have become available, making possible measurement and comparison of low CRP levels in blood. These sensitive assays have revealed the relationship between hs-CRP levels and the development and progression of coronary heart disease [23, 24] and osteoarthritis [31]. Moreover, the significant correlations between hs-CRP and diabetes [32] and airway diseases such as chronic obstructive pulmonary disease [33] and asthma [34] have been reported. In the present study, disease severity was correlated with hs-CRP in stable bronchiectasis, demonstrating that hs-CRP may be a good biomarker in low-grade systemic inflammation in such patients.

Bronchiectasis patients in a stable phase with elevated levels of systemic markers of inflammation have been studied [17]. Other authors [35] have suggested that even in periods of clinical stability, patients with non-CF bronchiectasis experience increased bronchial inflammation. During an exacerbation, particularly an infective episode, large quantities of neutrophils migrate into the airway, which can lead to increased levels of proteolytic agents. These agents participate in the destruction of the lung matrix and contribute to the development of bronchiectasis. The same authors [35] noted that, while the observed increase in inflammation during exacerbations decreases with antibiotic treatment, it does not disappear entirely. This may be the cause of the higher hs-CRP levels observed in our patients with multiple exacerbation-related hospitalizations.

Levels of CRP (not hs-CRP) have been shown to significantly correlate with HRCT bronchiectasis scores; however, a very poor correlation with lung function measures has also been reported [17]. In the current study, hs-CRP had a marginal, negative correlation with lung function measures (FVC or FEV_1_). This may be because there was only low-grade inflammation in these patients and the fact that the hs-CRP assay is more sensitive than the CRP assay. Twenty-nine patients (42%) had hs-CRP levels lower than 3.0 mg/L in this study.

The 6-minute walk test, a functional assessment of patients with cardiopulmonary disease, is a good outcome predictor of obstructive airway diseases such as COPD and idiopathic pulmonary fibrosis. However, it has been reported that exercise tolerance demonstrates a stronger correlation to health-related quality of life than physiological measures of lung function or disease severity in bronchiectasis [36]. Other exercise tests have been used to assess cystic fibrosis-related bronchiectasis in children [37], and the results revealed a poor correlation between exercise test and HRCT abnormalities. In the current study, there was no difference in walking distances between the hs-CRP groups, indicating a more complicated pathogenesis and disease progression in bronchiectasis than in other obstructive airway diseases.

Resting oxygen saturation was significantly lower in the higher hs-CRP group. Furthermore, the association between the need for long-term oxygen therapy and mortality has been reported [38]. The correlation between hs-CRP and baseline oxygenation saturation (rest O_2_%) may reflect the underlying disease activity in these patients. Moreover, the stronger correlation between rest O_2_% and HRCT (*r* = −0.478, *P* < 0.001) suggests that it can be a useful tool in assessing disease severity in stable bronchiectasis, although its role in disease progression and mortality warrants further investigations.

A high prevalence of atopy and increased serum ECP in adult patients with bronchiectasis has been reported [39, 40]. Serum ECP may be more relevant in assessing local eosinophil involvement than number of blood eosinophils. Atopic status was shown to not affect hs-CRP levels in steroid-naive or steroid-using asthma patients [34]. There was no significant difference between the hs-CRP groups in the current study, which suggests that eosinophils in the airway do not play a key role in persistent inflammation in bronchiectasis.

Bacterial infections are a major cause of morbidity and mortality in bronchiectasis patients. Acute inflammation is an important host defense against bronchial infection; however, if the infection becomes chronic, it can cause lung damage and lead to disease progression [3, 41]. The most common bacteria in the current study were *Pseudomonas aeruginosa*, which is consistent with previous reports [17]. Patients with bronchiectasis in a stable phase have raised levels of systemic markers of inflammation; however, this was not dependent on the presence of colonization in sputum in the current study.

Because there are so few randomized controlled trials of therapies for non-CF bronchiectasis and no US Food and Drug Administration approved therapies for non-CF bronchiectasis, patients must be evaluated and treated on an individual basis. Patients with mild-to-moderate bronchiectasis and infrequent exacerbations may not need maintenance therapy. According to the study of K.W. Tsang, inhaled corticosteroid (ICS) treatment is beneficial to patients with bronchiectasis, particularly those with *Pseudomonas aeruginosa* infection [42]. Twenty-three patients (35%) received regular inhaled cortical steroid/long acting *β*_2_ agonist (LABA) treatment in the current study; however, no patient was treated with long-term macrolides to prevent exacerbations. There are 4 patients (5.8%) with diabetes mellitus and 9 patients (13.0%) with positive methacholine provocation test in the current cohort study. Therefore, the inhaled corticosteroid/long acting *β*_2_ agonist (LABA) treatment was prescribed for our bronchiectasis patients, either with or without airway hyperreactivity/asthma. There was no significant difference between the hs-CRP groups in the present study (data not shown), which may reflect the minor role of such therapy in systemic inflammation in stable bronchiectasis. Interestingly, none of the other management strategies applied in our cohort, including therapy with inhaled and oral steroids, antibiotics, and secretion clearance maneuvers and oxygen therapy, had a significant effect on hs-CRP. Previous study showed that in patients with asthma on ICS treatment, serum hs-CRP levels did not differ from those of healthy controls and did not correlate with clinical or sputum indices. It is likely that the ICS, which has well-characterized anti-inflammatory properties, used in these patients might have reduced serum hs-CRP [34]. Similarly, there is no previous study to confirm the effects of ICS/LABA on the serum level of hs-CRP in bronchiectasis. Further research will be considered to study this important issue.

Two patients died of pneumonia and respiratory failure later within the study period, and their initial hs-CRP levels were 18.78 and 18.81 mg/L, respectively. Hence, the significance of higher hs-CRP in stable bronchiectasis needs further investigation.

There are several limitations to the current study. First, the number of patients is limited, and they were recruited from a single hospital, which may limit the generalizability of the study results. Second, evolutionary variables such as clinical evolution and the numbers of following exacerbation or hospitalization were not included in the analysis due to the short study period. Third, important transversal variables related to bronchiectasis such as systemic inflammatory diseases other than cardiovascular disorders and quality of life were not included in the analysis because the limited number of patients did not allow for the inclusion of more variables in the factorial analysis. Only four patients had type 2 diabetes mellitus and the study of osteoporosis was incomplete. Finally, the impact of nontuberculosis mycobacterial colonization or infection in these patients was not studied. Therefore, larger, multicentric studies are needed, with long-term follow-up and a larger number of patients in order to corroborate our results.

In conclusion, in patients with stable non-CF bronchiectasis, there was a good correlation between serum hs-CRP and HRCT scores. Increased HRCT scores and decreased rest oxygenation saturation were associated with higher levels of serum hs-CRP, which suggests that serum hs-CRP may be a useful biomarker that directly reflects the degree of systemic inflammation in stable non-CF bronchiectasis. However, further studies are required in order to better elucidate the clinical significance of the role of hs-CRP in bronchiectasis progression and treatment response, either in anti-inflammatory pharmacological therapy or in regular pulmonary rehabilitation programs.

## Figures and Tables

**Table 1:** Demographic data of the 69 stable bronchiectasis patients.

Demographic factor	Mean (SD)	95% CI
Age (years)	57.5	54.2–60.8
BMI (kg/m^2^)	22.0	21.2–22.8
FVC (L)	2.1	1.9–2.3
FVC% predicted	67.4	62.7–72.0
FEV_1_ (L)	1.5	1.4–1.7
FEV_1_% predicted	62.6	57.1–68.1
6 MWD (m)	454.4	432.4–476.4
Rest O_2_S%	95.1	94.3–95.9
HRCT score	26.2	23.1–29.3
hs-CRP (mg/L)	4.5	3.6–5.5

Abbreviations: BMI: body mass index, FVC: forced vital capacity, FEV_1_: volume that has been exhaled at the end of the first second of forced expiration, 6 MWD: 6-minute walk test distance, HRCT: high-resolution computed tomography, and hs-CRP: high-sensitivity C-reactive protein.

**Table 2:** Characteristics and outcomes of the 69 stable bronchiectasis patients.

	hs-CPR < 4.26	hs-CRP ≥ 4.26	*P* value
	*n* = 38	*n* = 31	
Age (years)	56.2 ± 13.7	59.0 ± 13.9	0.406
Gender (M/F)	21/17	18/13	0.815
BMI (kg/m^2^)	22.0 ± 3.4	22.0 ± 3.4	0.990
Smoking			
Never	30	25	0.862
Ex/current	8	6	
PFT			
FVC (L)	2.25 ± 0.81	1.85 ± 0.71	0.034
FVC (%)	72.5 ± 16.4	61.1 ± 21.0	0.014
FEV_1_ (L)	1.69 ± 0.74	1.34 ± 0.59	0.038
FEV_1 _(%)	67.6 ± 21.4	56.6 ± 23.5	0.046
FEV_1_/FVC (%)	73.5 ± 11.0	71.3 ± 10.4	0.401
Total IgE (KU/L)	137.8 ± 320.3	170.9 ± 490.6	0.737
ECP (mcg/L)	17.5 ± 17.8	24.5 ± 36.7	0.304
6 MWT			
Rest O_2_ sat (%)	96.4 ± 1.6	93.5 ± 4.4	0.001
Lowest O_2_ sat (%)	87.7 ± 6.4	85.2 ± 10.2	0.237
ΔO_2 _sat (%)	8.7 ± 6.1	8.4 ± 7.4	0.816
Walk distance (meters)	469.3 ± 78.2	436.1 ± 104.1	0.136
HRCT scores	21.7 ± 9.8	28.1 ± 13.1	0.004
Bacterial colony			
*Ps. aeruginosa *	6	11	0.115
Others	8	4	
Normal flora/no growth	24	16	
Hospitalizations before recruitment (times/year)			
<2	35	21	0.01
≥2	3	10	

Abbreviations: hs-CRP: high-sensitivity C-reactive protein, PFT: pulmonary function test, IgE: immunoglobulin E, ECP: eosinophilic cationic protein, 6 MWT: 6-minute walk test, ΔO_2_ sat (%): oxygenation difference between rest and lowest during 6-minute walk test, and HRCT: high-resolution computed tomography.

**Table 3:** Correlations between hs-CRP, clinical variables, and HRCT score.

	hs-CRP(mg/L)	*P* value
Age (years)	0.124	0.312
BMI (kg/m^2^)	−0.094	0.441
FVC (L)	−0.161	0.187
FEV_1_ (L)	−0.153	0.211
FEV_1_/FVC	−0.058	0.637
IgE (KU/L)	0.180	0.140
ECP (mcg/L)	0.087	0.479
Rest O_2_%	−0.269	**0.025**
Lowest O_2_%	−0.108	0.376
ΔO_2_%	−0.003	0.982
6 MWD (m)	−0.190	0.118
HRCT score	0.473	<**0.001**

Abbreviations: BMI: body mass index, FVC: forced vital capacity, FEV_1_: first second, 6 MWD: 6-minute walk test distance, HRCT: high-resolution computed tomography, and hs-CRP: high-sensitivity C-reactive protein.

## Abbreviations

Hs-CRP: High-sensitivity C-reactive protein
CF: Cystic fibrosis
PFT: Pulmonary function test
IgE: Immunoglobulin E
ECP: Eosinophilic cationic protein
6 MWT: 6-minute walk test
HRCT: High-resolution computed tomography
ICS: Inhaled steroids
LABA: Long-acting beta-agonists.
